# Supplementary material for: Large-scale classification of metagenomic samples: a comparative analysis of classical machine learning techniques vs a novel brain-inspired hyperdimensional computing approach
Source: bioRxiv. 2025 Dec 2:2025.07.06.663394. Originally published 2025 Jul 7. Preprint. [Version 2] doi: 10.1101/2025.07.06.663394 (PMC12265723; doi:10.1101/2025.07.06.663394)
Supplement: Supplement 3 [file NIHPP2025.07.06.663394v2-supplement-3.pdf]

bioRxiv preprint doi: https://doi.org/10.1101/2025.07.06.663394; this version posted December 2, 2025. The copyright holder for this preprint (which was not certified by peer review) is the author/funder, who has granted bioRxiv a license to display the preprint in perpetuity. It is made available under aCC-BY 4.0 International license.

Supplementary Material

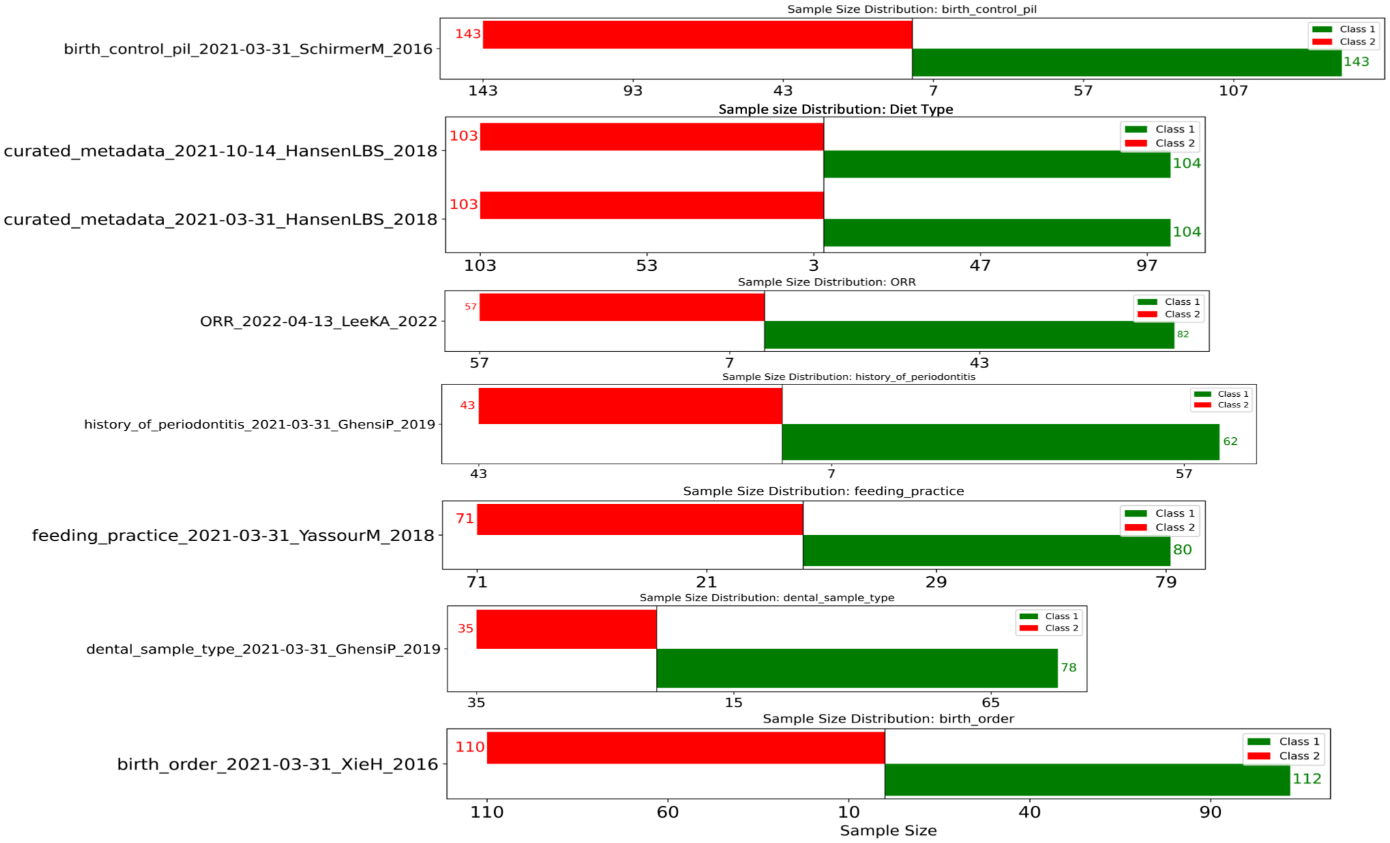

Figure S1. Sample distribution across the class labels: birth control pill vs. no birth control pill use.

bioRxiv preprint doi: https://doi.org/10.1101/2025.07.06.663394; this version posted December 2, 2025. The copyright holder for this preprint (which was not certified by peer review) is the author/funder, who has granted bioRxiv a license to display the preprint in perpetuity. It is made available under aCC-BY 4.0 International license.

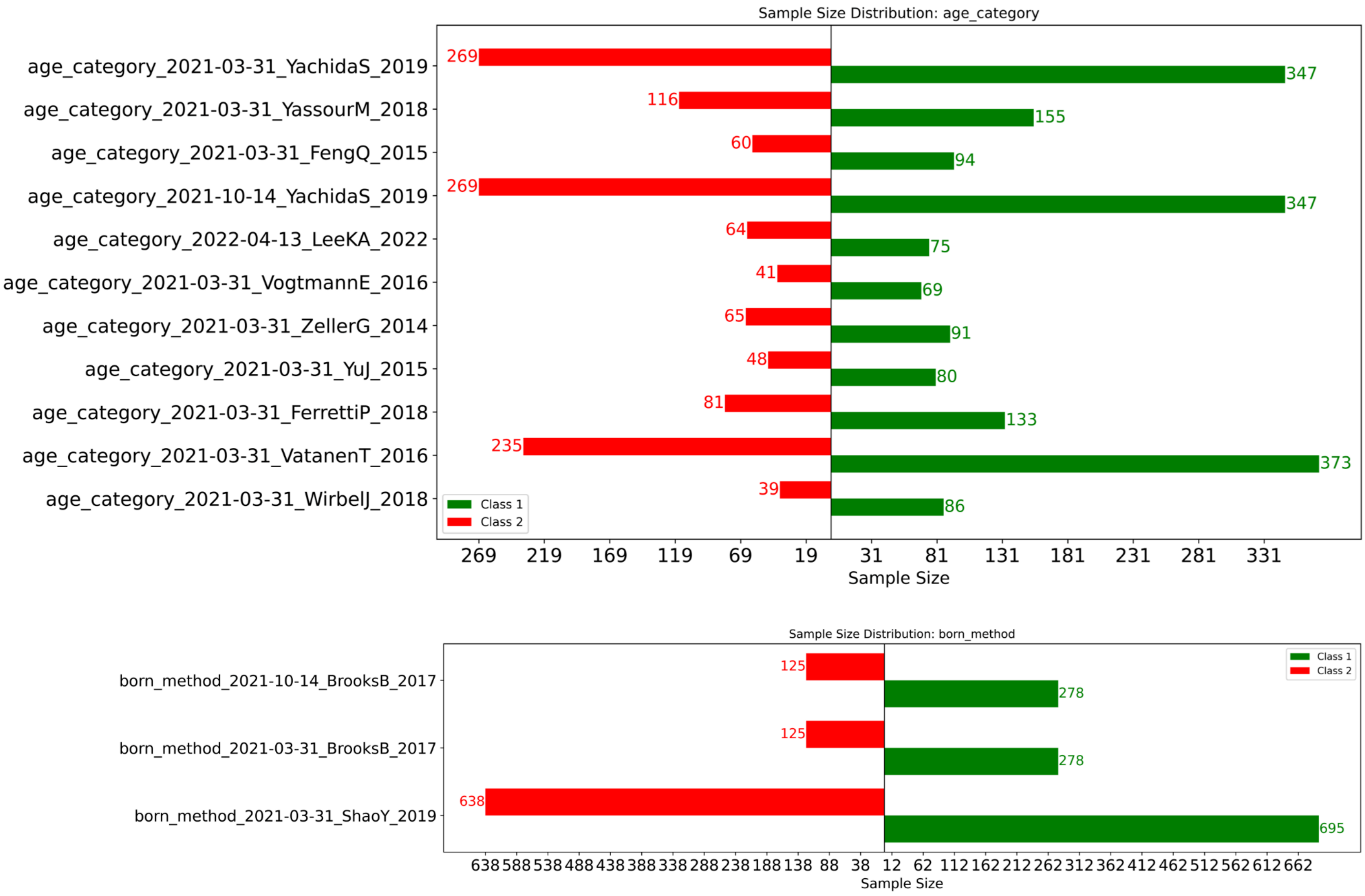

Figure S2. Sample distribution across both class labels in two different categories: age, where the class labels are young vs. old, and birth method, where the class labels are normal delivery vs. C-section.

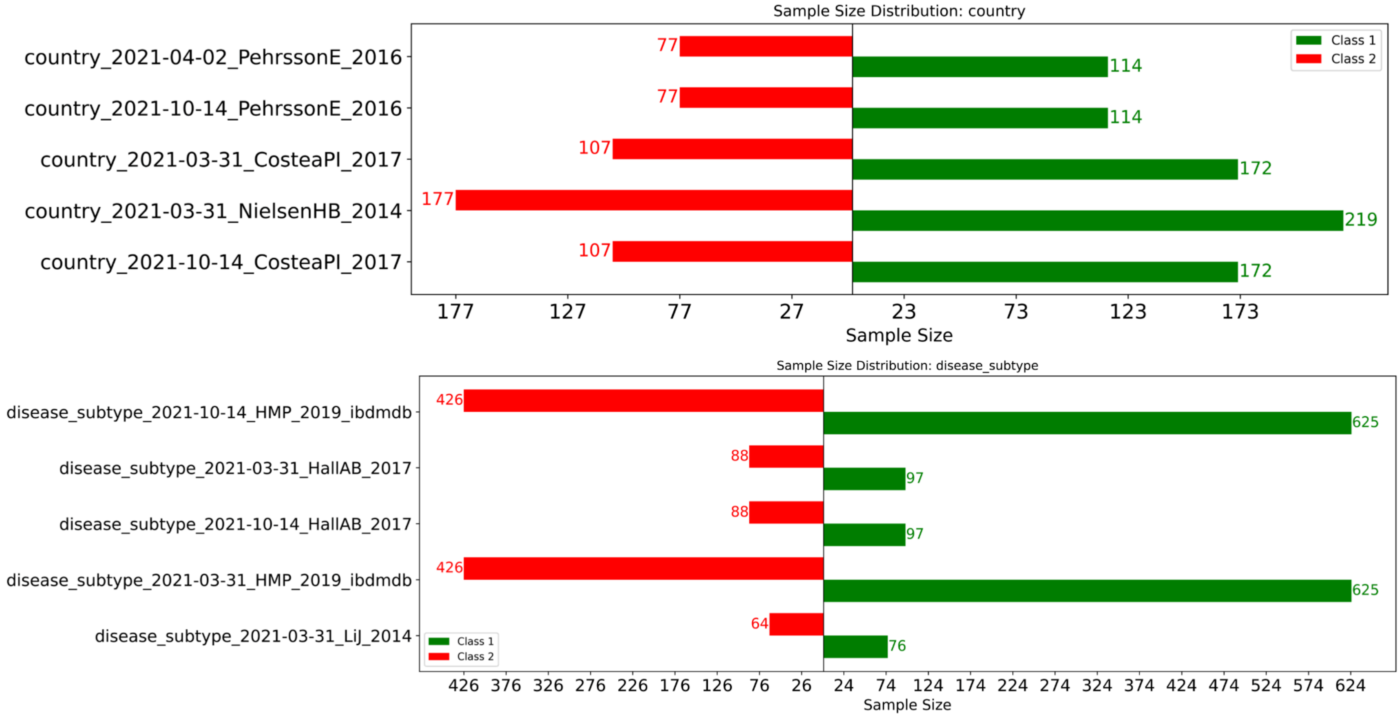

bioRxiv preprint doi: https://doi.org/10.1101/2025.07.06.663394; this version posted December 2, 2025. The copyright holder for this preprint (which was not certified by peer review) is the author/funder, who has granted bioRxiv a license to display the preprint in perpetuity. It is made available under aCC-BY 4.0 International license.

Figure S3. Sample distribution across two different categories: **country of origin**, where the class labels represent two different countries, and **disease subtype** where the class labels represent two different disease subtypes (e.g., Irritable Bowel Disease, Crohn's Disease, or Diabetes Type I vs. Type II).

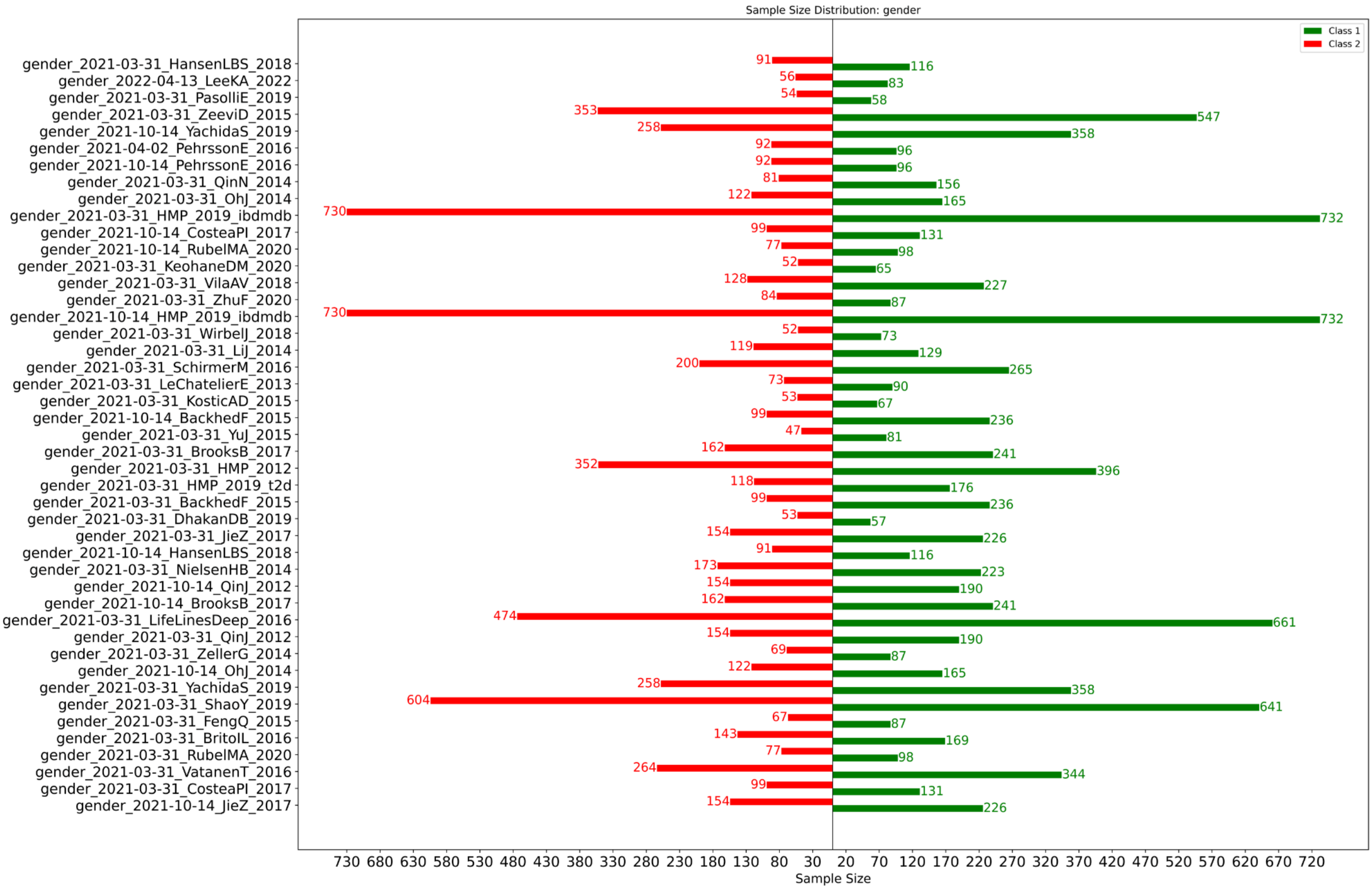

Figure S4. Sample distribution across both class labels male vs female.

bioRxiv preprint doi: https://doi.org/10.1101/2025.07.06.663394; this version posted December 2, 2025. The copyright holder for this preprint (which was not certified by peer review) is the author/funder, who has granted bioRxiv a license to display the preprint in perpetuity. It is made available under aCC-BY 4.0 International license.

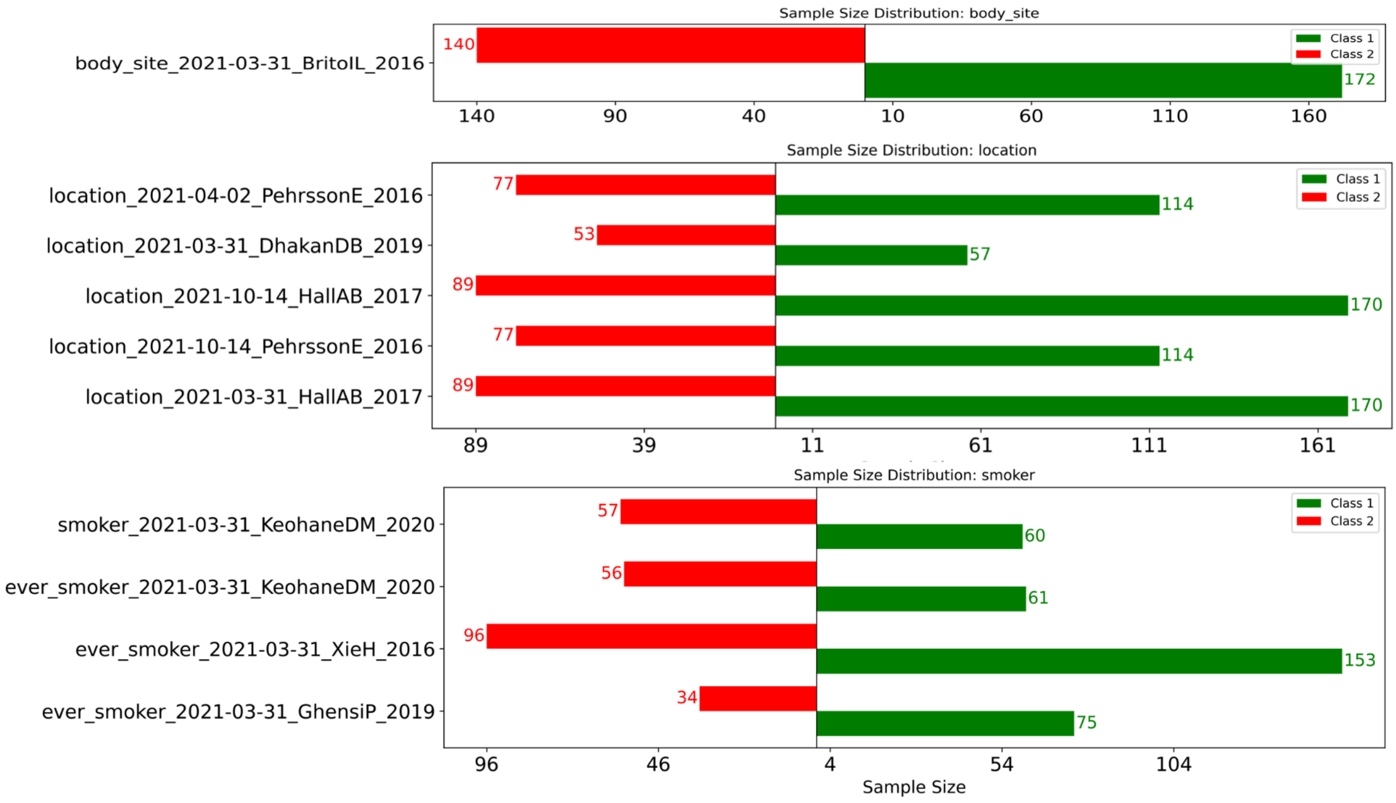

Figure S5. Sample distribution across three different categories: body site, where the class labels represent the different body parts where the samples were collected (e.g., skin, saliva, or feces); location, where the class labels correspond to two different cities; and smoking status, where the class labels are smoker vs. non-smoker.

bioRxiv preprint doi: https://doi.org/10.1101/2025.07.06.663394; this version posted December 2, 2025. The copyright holder for this preprint (which was not certified by peer review) is the author/funder, who has granted bioRxiv a license to display the preprint in perpetuity. It is made available under aCC-BY 4.0 International license.

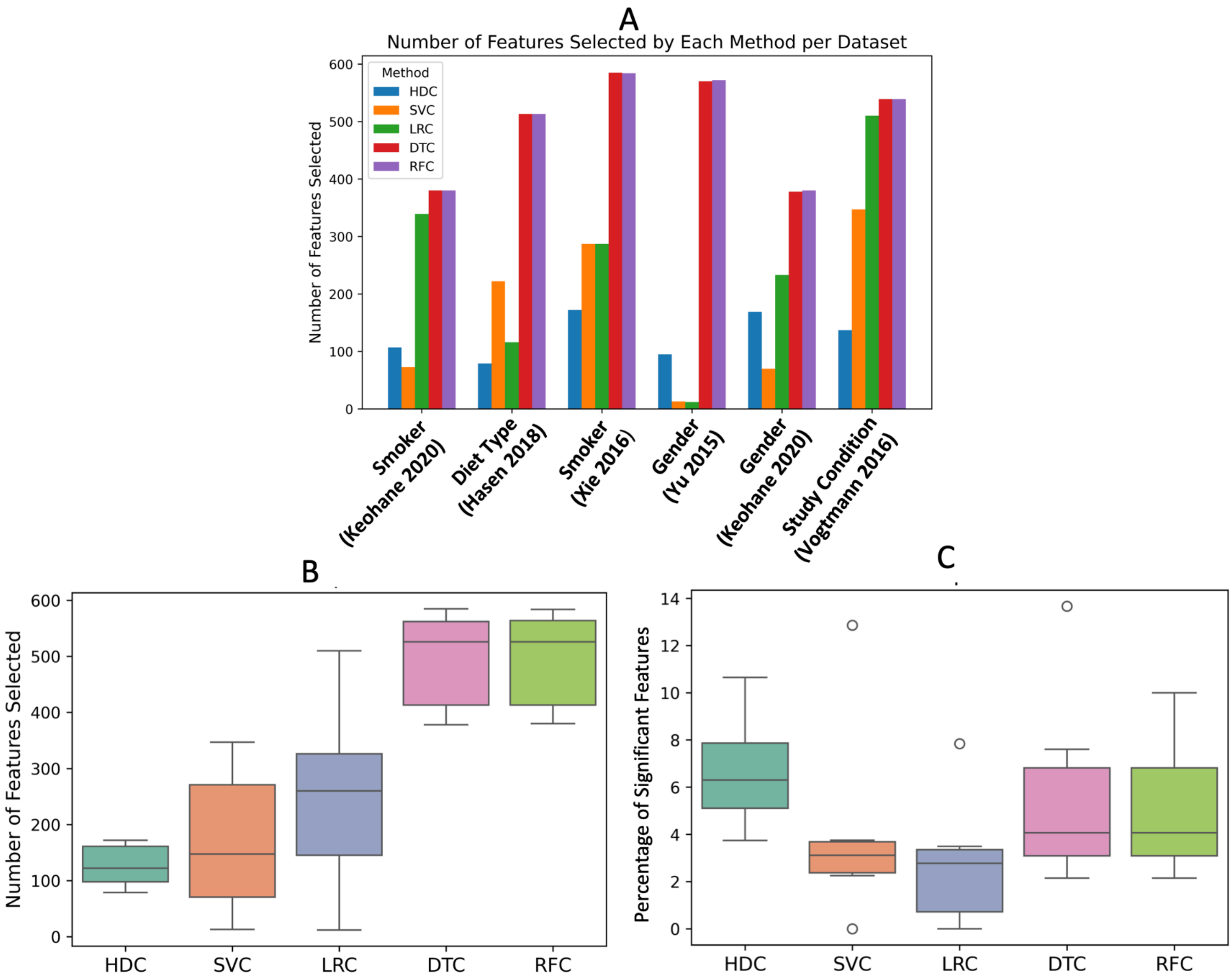

Figure S6. Panel A: The bar plot represents the number of selected features by each algorithm across the datasets for which HDC performs better in comparison to all the other algorithms. Panel B: The box plot shows the distribution of selected features across the same datasets considered in Panel A, where HDC outperformed all other algorithms. Panel C: Distribution of the percentage of significant features among selected features calculated based on the Wilcoxon Rank-Sum Test.
